# Advanced microfluidic and 3D cell culture platforms for modeling vascularization in diabetic foot ulcers: A systematic review of translational challenges and perspectives

**DOI:** 10.1371/journal.pone.0328278

**Published:** 2026-04-06

**Authors:** Ana Karoline Almeida da Silva, Gustavo Adolfo Marcelino de Almeida Nunes, Rafael Mendes Faria, Klériston Santos, Rafael Pissinati de Souza, Lindemberg Barreto Mota da Costa, Marcos Augusto M. Fonseca, Sheila Sousa Gomes Fortes, Amanda Maciel Lima, Isolda Monteiro, Pedro Henrique Almeida, Ana Carolina Migliorini Figueira, Harsson S. Santana, Marcella Lemos Brettas Carneiro, Glécia Virgolino da Silva Luz, Graziella Joanitti, José Carlos Tatmatsu-Rocha, Mário Fabrício Fleury Rosa, Maria Alice Martins, Ícaro Santos, Emanuel Carrilho, Adson Ferreira da Rocha, Suélia de Siqueira Rodrigues Fleury Rosa

**Affiliations:** 1 University of Brasilia - UnB, Campus Darcy Ribeiro, Postgraduate Programme in Mechatronic Systems, Mechanical Engineering Department, Brasilia, Distrito Federal, Brazil; 2 Federal Institute of Education, Science and Technology of Triângulo Mineiro - IFTM, Campus Paracatu, Department of Electrical Engineering, Paracatu, Minas Gerais, Brazil; 3 Federal Institute of Education, Science and Technology of Rondônia, Porto Velho, Rondônia, Brazil; 4 University of Brasilia - Postgraduate Program in Biomedical Engineering-PPGEB, Faculty of Engineering Sciences and Technologies - FCTE, Gama, Distrito Federal, Brazil; 5 University of São Paulo - USP, Institute of Chemistry of São Carlos, Department of Chemistry and Molecular Physics, São Carlos, São Paulo, Brazil; 6 University of São Paulo - USP, Institute of the Sea, Department of Bioproducts and Bioprocesses, Santos, São Paulo, Brazil; 7 Federal University of Ceará – UFC, Physical Therapist, Center for Research and Technological Innovations in Human Rehabilitation – INOVAFISIO, Fortaleza, Ceará, Brazil; 8 National Center for Research in Energy and Materials - CNPEM, National Laboratory of Biosciences - LNBIO, Campinas, São Paulo, Brazil; 9 Renato Archer Information Technology Center, Campinas, São Paulo, Brazil; 10 University of Brasilia - UnB, Campus Ceilândia, Postgraduate Program in Nanoscience and Nanobiotechnology and Postgraduate Program in Health Sciences and Technologies, Ceilândia, Distrito Federal, Brazil; 11 Federal University of Ceara – UFC, Medicine School, Postgraduate Programs in Biomedical Engineering and in Physiotherapy and Functionality, Fortaleza, Ceara, Brazil; 12 Nanotechnology National Laboratory for Agriculture, Embrapa Instrumentation, São Carlos, São Paulo, Brazil; 13 University of Wisconsin-Madison, Department of Biomedical Engineering, Madison, Wisconsin, United States of America; 14 National Institute of Science and Technology in Bioanalytics - Lauro Kubota, Campinas, São Paulo, Brazil; 15 Meinig School of Biomedical Engineering, Master of engineering (M.Eng.) Program, Cornell University, Ithaca, New York, United States of America; KIST: Korea Institute of Science and Technology, GERMANY

## Abstract

The healing process of diabetic foot ulcers (DFUs) presents a slow pattern with an increased risk of infections, ischemia, and thrombosis correlated with high levels of reactive oxygen species production. Vascular injury is one of the factors contributing to the difficulty of wound healing in diabetic patients. Although the understanding of the pathophysiology of DFUs has significantly increased in recent years, associated treatments still have a high level of failure, leading to high morbidity rates, mortality, and amputations. Three-dimensional (3D) cell culture platforms offer a new approach to investigating and treating these wounds, as they can reproduce one or more physiological systems in a relevant microenvironment. This systematic review describes the advancements, challenges, and future implications of advanced 3D culture models in vascularization, encompassing pathophysiological understanding, treatment, and prognostic perspectives. We followed the Preferred Reporting Items for Systematic Reviews and Meta-Analyses (PRISMA) guidelines. This work was registered under PROSPERO protocol number CRD42022336473. The eligibility criteria addressed studies related to chronic DFUs that analyzed vascularization during the healing process. The selected study designs involved 3D cultures and organ-on-a- chip (OoC) platforms, utilizing either primary or secondary human cell lines. Studies published more than 10 years ago or using only animal cells in 2D culture environments were excluded. The search was conducted in PubMed, LILACS, Embase, MEDLINE, IEEE, the BSV regional portal, ScienceDirect, Scopus, CINAHL, EBSCO, and Web of Science. A total of 2,539 relevant studies were identified as of June 1, 2025. After screening, only seven met the inclusion criteria. These studies reflect a growing interest in using hydrogel scaffolds and microfluidic systems to replicate diabetic skin environments; however, the field remains in an early stage of development. OoC platforms, in particular, stand out for their ability to recreate dynamic, tissue-like conditions and vascular function. While some promising attempts have been made to combine hydrogels with these technologies, the evidence is still limited and inconclusive for chronic wound modeling. This review underscores both the potential and the urgent need for more robust and translational research in this area—especially toward building personalized, clinically relevant models to support future drug testing and therapeutic innovation.

## Introduction

Diabetes mellitus (DM) is a disorder of the endocrine system characterized by high blood glucose levels and can lead to both microvascular and macrovascular dysfunctions [1]. Among its most severe complications are diabetic foot ulcers (DFUs), which affect up to 34% of individuals with DM during their lifetime. Failures in the healing process of these wounds frequently result in amputations, with approximately half of all lower-limb amputations in diabetic patients caused by infected ulcers. Alarmingly, the mortality rate after amputation is high, with around one-third of patients dying within one year and nearly two-thirds within four years [2].

In addition to physical deterioration, DFUs significantly reduce patients’ quality of life and are associated with psychological and familial burdens [3]. Global healthcare spending related to diabetes was estimated at $700 billion in 2019 and is projected to rise to $825 billion by 2030, with nearly one-third of these costs attributed to DFU care [4]. The healing of tissue injuries involves multiple, tightly regulated phases—hemostasis, inflammation, proliferation, and remodeling—all of which can be disrupted in chronic wounds. In DFUs, wound healing is delayed and heterogeneous, increasing susceptibility to infection, ischemia, and thrombosis. Chronic hyperglycemia, a hallmark of diabetes, is closely associated with impaired wound healing, atherosclerosis, and neuropathy.

Persistent hyperglycemia causes endothelial dysfunction, inhibits the migration of keratinocytes and fibroblasts, and induces oxidative stress through the overproduction of reactive oxygen species (ROS). These effects contribute to vascular injury, reduced vasculogenesis, and an inflammatory milieu that impairs tissue regeneration [1,5,6]. Even in the absence of hyperglycemia, associated conditions such as obesity and hypertension exacerbate endothelial damage. Multiple factors contribute to the poor prognosis of DFUs, including genetic predispositions, metabolic imbalances, and inflammatory cascades [7]. Traditional preclinical models fail to replicate the complexity of these conditions, often lacking dimensionality, disease-specific cellular interactions, and immune crosstalk [8].

To overcome these limitations, there is a growing interest in alternative experimental methods that move beyond animal testing. In vivo studies often lack reproducibility due to physiological and genetic differences between species. Ethical considerations and translational limitations have led to the global advancement of animal-free testing protocols. The European Union, the United States, Japan, and Brazil have each promoted initiatives supporting alternative models, such as the National Network of Alternative Methods (RENAMA) in Brazil [9–11].

Emerging technologies such as organ-on-a-chip (OoC) systems are now enabling the simulation of vascular and systemic interactions involved in DFUs. Ji et al. (2023) demonstrated that apolipoprotein A-IV (Apo A-IV), isolated from DFU patients, promotes TNF-*α* expression in microfluidic arterial models, suggesting a direct contribution of systemic inflammation to chronic wound persistence [12]. Out-of-chamber (OoC) platforms replicate dynamic flow and mechanical stress conditions, providing mechanistic insights into endothelial dysfunction and immune dysregulation.

Considering the biological variability among patients and the limitations of conventional therapies, 3D cell culture platforms have emerged as promising tools for studying DFUs. These platforms mimic native tissue architecture, allowing for the exploration of complex interactions at cellular, molecular, and biophysical levels. They also facilitate modeling interactions between diseased and heterotypic cell types—such as keratinocytes, fibroblasts, and macrophages—enabling more accurate and predictive preclinical studies [13–16].

Traditional models such as 2D cultures and animal assays are insufficient to replicate the chronic inflammatory microenvironment, immune dysfunction, and extracellular matrix (ECM) degradation observed in DFUs [17]. In contrast, DFU-on-a-chip platforms integrate pathophysiological features using patient-derived cells embedded in biomimetic scaffolds that support endogenous diseased ECM production. When combined with microfluidic flow, these systems reproduce gradients, mechanical stimuli, and immune cell recruitment patterns similar to those found in in vivo environments, providing robust platforms for testing topical therapies and personalized treatments [18].

Recent studies emphasize the importance of incorporating immune-competent models into OoC systems. By using patient-derived keratinocytes, fibroblasts, and macrophages, researchers have recapitulated the persistent inflammation and delayed healing characteristics of DFUs. Analogously, in diabetic nephropathy (DN), microfluidic glomerulus-on-a-chip devices have replicated features such as podocyte detachment and oxidative stress under hyperglycemic conditions—highlighting the need for dynamic, multicellular platforms in diabetes research [19].

Additionally, 3D systems such as nitric oxide (NO)-loaded hydrogels offer therapeutic innovations. These biomaterials mimic ECM structure, support realistic cellular interactions, and allow localized, controlled drug release. While OoC systems serve predominantly in mechanistic research, 3D hydrogels—such as PNO gels—have demonstrated translational potential for tissue regeneration, angiogenesis, and inflammation modulation in DFU treatment [20–22].

Hydrogels, with their high water content and biocompatibility, closely resemble biological tissues and are suitable for creating DFU-relevant microenvironments [17,23]. In OoC systems, they act as scaffolds for 3D cultures with microchannels and chemical gradients. Common materials used include alginate, collagen, agarose, and polyethylene glycol (PEG), which are applied via bioprinting, sacrificial molding, or layer-by-layer assembly. These platforms allow fine-tuned studies of chemotaxis and angiogenesis under chronic inflammation [24].

Given the multifactorial nature of DFUs—characterized by chronic inflammation, poor vascularization, and impaired remodeling—they serve as an ideal model for the development of 3D and OoC platforms. These technologies not only improve our understanding of disease mechanisms but also hold promise for the development of regenerative therapies tailored to real human pathology, offering new hope for patients affected by this debilitating complication.

This systematic review outlines the main advances in microfluidic modeling and highlights the challenges and potential impacts on understanding, treating, and prognosticating diabetic foot wounds. These insights may inspire future innovations and translational ventures.

## Materials and methods

### Protocol and registration

We registered this systematic review on the International Prospective Register of Systematic Reviews (PROSPERO) under registration number CRD42022336473. We followed the guidelines of the Preferred Reporting Items for Systematic Reviews and Meta-Analysis for this review [25].

### Eligibility criteria

*Inclusion criteria.*

The inclusion criteria for this systematic review followed the population, intervention, comparison, outcome, and study design (PICOS) approach [26]. The selected studies involved diabetic foot wounds (P). They examined impaired vascularization during the healing process (I) using human primary and secondary lineage cell cultures (C) in a 3D cell culture setting (O) through in vitro studies using an OoC platform or 3D culture (S).

*Exclusion criteria.* We excluded studies that did not meet the inclusion criteria based on the PICOS approach. Specifically, studies that did not involve DFU type wounds, did not analyze impaired vascularization during the healing process, did not use human primary and secondary lineage cell cultures, did not involve a 3D cell culture setting, or did not use an OoC platform were excluded . We also excluded studies that utilized pluripotent or primary cells from animals. The exclusion criteria were prioritized in the following order:

1. studies that did not provide information on vascularization or wound healing
2. studies using donor cells not from living humans or cadavers
3. studies consisting of reports, letters, personal opinions, book chapters, conference summaries, and patents
4. studies using donated animal cells
5. studies not done in vitro and randomized clinical trials on humans
6. studies published over 10 years ago

### Search strategy and information sources

The review question was as follows: What is the application of OoC in vascularizing chronic wounds in people with diabetes? Search strategies were developed and adapted according to the needs of each of the databases used for research: PubMed, LILACS, Embase, MEDLINE, IEEE, regional portal BSV, ScienceDirect, Scopus, CINAHL EBSCO , and Web of Science. We searched databases published in the last 10 years, from January 2023 until June 2025, with no language restrictions. Duplicate references were removed using *Rayyan*^®^ software [27]. The results are presented in Supplementary Material section.

### Study selection and data extraction

In the first phase, we screened the titles and abstracts of all references identified in the electronic databases and selected articles that met the inclusion criteria. The selections of the first phase were made by peers using the *RayyanRayyan*^®^ software [27], applying the predefined inclusion and exclusion criteria. In the second phase, we independently analyzed the full texts in pairs and picked papers using a standardized data extraction form. If there were disagreements, a third author was consulted. This process was implemented to minimize discrepancies and bias between reviewers.

The relevant data from each study were extracted and organized in a predefined table that described the main points related to the characteristics of the intervention of interest, including the type of cell donor, cell lineage, data on microfluidic simulation, and angiogenesis rate, and its relationship with chronic wound healing in diabetic patients.

### Risks of bias and quality in individual studies

Due to the heterogeneity of the evaluated interventions, it was not possible to perform a meta-analysis of all the data related to the findings. Therefore, we analyzed the methodological quality of the studies using the Cochrane Robvis Collaboration Tool (Rob 2), which we adapted as described below. We performed evaluations individually and in pairs, and we called a third team member to mediate in cases of disagreement [28,29].

Bias risk analysis using the RoB 2 platform is a fundamental step in critically evaluating the methodological quality of primary studies. It can assist in the interpretation of the results of systematic reviews and meta-analyses. However, a specific tool for evaluating the methodological quality of in vitro 3D trials is currently needed [30].

We adapted an available version of the RoB 2 tool to create 15 domains to assess the methodological quality of the OoC assays. These domains cover various aspects, including selection of tissues and cells, presence of experimental and control groups, identification and authenticity of the cell lines, basic morphological descriptions, allocation of tissues or cells, steps of the cell layer formation procedures, pre-established criteria for inclusion or exclusion of experimental units, blinding of evaluators, loss of follow-up, standardized data collection, detailed description of chip design platform development, submission to research ethics committees, control of confounding factors, and declaration of conflicts of interest. These domains can be used to assess the risk of bias in OoC studies and to determine their overall quality. By carefully evaluating each part, one can decide whether the risk of bias is low or high in each study.

Studies rated as “LOW” showed sufficient evidence of appropriate measures to reduce bias. This suggests that the risk of bias is low and the results are reliable. Those rated “NO INFORMATION” provided insufficient information for assessing the risk of bias. This indicates that more data are needed for a proper evaluation. Those rated “CRITICAL” were associated with a high risk of bias that could affect the validity of the results. This indicates that the study limitations are significant and may compromise the reliability of the findings. Those rated “HIGH” had a high risk of bias, which could impact the interpretation of the results. This suggests that the study limitations are substantial and should be considered when analyzing the findings. Those rated “UNCLEAR” provided insufficient or ambiguous information for determining the risk of bias. This indicates that the assessment of bias cannot be made with certainty due to a lack of clear information.

The outcomes that reviewers of the articles raised were measured and used to classify the studies based on the percentage of “YES” responses to the criteria. Studies with ≥ 70 % “YES” answers were considered high quality, studies with 50-69% “YES” responses were regarded as moderate quality, and studies with ≤ 49% “YES” responses were considered low quality.

## Results

**Fig 1:**
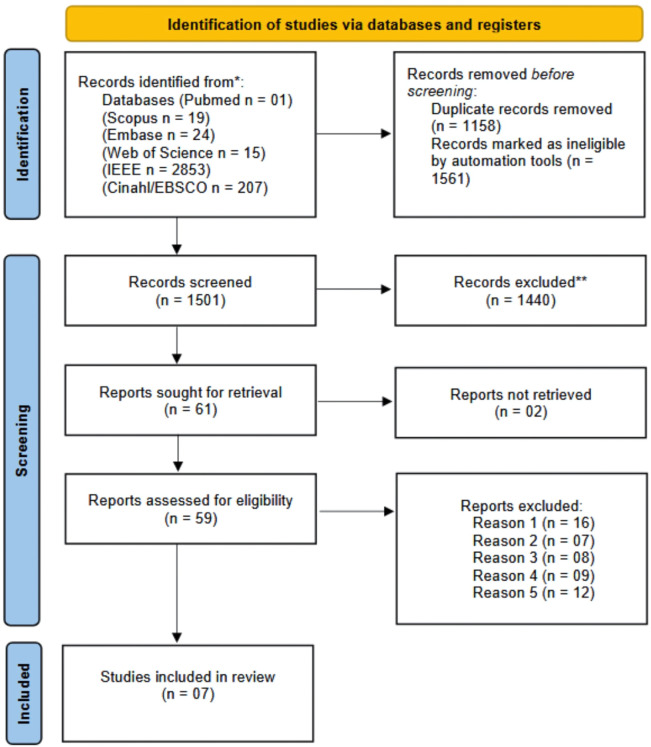
PRISMA flow diagram of the study selection process in the systematic review. The diagram shows the number of records identified, screened, assessed for eligibility, and included in the review, based on the defined inclusion and exclusion criteria. The process followed the PRISMA (Preferred Reporting Items for Systematic Reviews and Meta-Analyses) guidelines to ensure transparency and reproducibility.

**Fig 2:**
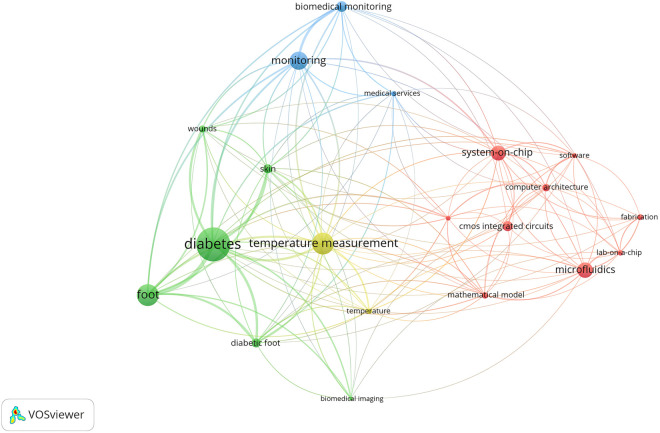
Bibliometric Analysis: Word Map - Bibliometric analysis was conducted using the VOSviewer program version 1.6.17 [32] on 1,343 works published with the search string (“Lab.-On-A-Chip Devices" OR “Body on a chip" OR “Microfluidics" AND “Angiogenesis Inducing Agents" OR “Angiogenesis Modulating Agents" OR “Neovascularization Physiologic" AND “Wound Healing" OR “Cicatrix" AND “Diabetes Mellitus" OR “Diabetic Foot") in the title, abstract, or keywords on January 24, 2023. The analysis used a minimum co-occurrence of terms of 30 times and binary counting.

Our systematic review aimed to investigate advances related to 3D cell culture and OoC platforms in the context of diabetic foot diseases. As shown in the PRISMA flow diagram, in Fig 1, the more notable contribution from scientific papers was found in electronic databases: Scopus (n = 19), Web of Science (n = 15), PubMed (n = 01), Embase (n = 24), and IEEE (n = 2853), totaling 3119 articles (Fig 2). After applying filters and removing duplicates, 1501 articles passed through the first filter of the review, which involved analyzing the titles and abstracts of the works. After the studies were selected, 61 papers were chosen for full-text reading. Despite a comprehensive search and recent advances in the microfabrication of microfluidic devices, only seven studies met the inclusion criteria and were selected to compose this review [17,23,31].

### Synthesis of the results

In a bibliometric study conducted on January 24, 2023, using VOSviewer software version 1.6.17, we searched the IEEE, PubMed, Scopus, and Embase databases using the MeSH search terms “lab-on-a-chip devices,” “microfluidics,” “angiogenesis,” and “diabetic foot.” We found 1343 articles, excluding duplicates. Fig 2 shows the most frequent keywords found in the articles, divided into four different groups: microfluidics (red), biomedical monitoring (blue), diabetes (green), and temperature measurement (yellow). The lines connecting the terms represent the co-occurrence relationships between them across the analyzed papers. The lack of studies focusing on 3D cell culture in the context of chronic wounds such as DFUs is evident. Some of the most frequently occurring terms in this field include diabetes, diabetic foot, temperature measurement, skin, wounds, biomedical monitoring, mathematical modeling, and microfluidics. The co-occurrence threshold for these terms was set at 20 or more occurrences (see Fig 2).

Despite being the target of this review, the studies found needed to present a DFU model on an OoC platform. However, the cultures were performed on other 3D structures using two distinct and complementary approaches, which may indicate paths for application using OoCs. These studies were recently developed and focused on the immune response of the skin, topical drug testing [17], ), and gene expression, as well as the response of diabetic skin to different stimulation [23].

[33] developed a tissue-specific multiple-organ-on-a-chip platform that emulates the pathophysiological conditions of Type 2 Diabetes Mellitus (T2DM) using 3D cell printing. Published in Advanced Functional Materials, the study integrates pancreatic islet, liver, and vascular tissue modules into a microfluidic system, allowing for dynamic crosstalk and systemic modeling of T2DM. The chip was fabricated using 3D bioprinting to deposit human-derived cells in organ-specific architectures, maintaining functionality across organs for over two weeks. Notably, the model recapitulated key features of T2DM, including insulin resistance, hyperglycemia, and endothelial dysfunction, which were validated through metabolic assays and histological analyses. Notably, the vascular component exhibited impaired angiogenic responses and increased expression of inflammatory markers under diabetic conditions.

Both studies performed cellular cultivation using a 3D gel-based setting (Table 1). Hydrogels, which are polymers with substantial water content, were employed. When cultivating 3D cells using hydrogels, it is crucial first to consider the mechanical resilience and porosity of the gels, given their diverse physical, biological, and chemical properties [17,23]. The mechanical durability of the device plays a significant function in fostering improved tissue organization and attachment to the framework.

**Table 1:** Summary of descriptive characteristics of the included studies and outcomes data.

Study	In vitro Assays	Intervention		Fabrication		Data Analysis	Outcomes
*Reference*	*Sample*	*Objective*	*Methodology*	*Formation of a cell layer*	*Cell culture methodology*	*Types of Analysis*	*Results*
[12]	Human arterial endothelial cells from microfluidic models	Explore the inflammatory effect of Apo A-IV from diabetic foot patients in microfluidic models	Apo A-IV glycosylated protein was applied to the arterial endothelium under dynamic conditions to evaluate TNF-*α* upregulation via NR4A3 signaling	Formation of endothelial layers in microfluidic chips mimicking arterial flow	Dynamic perfusion of cell culture medium through microchannels to simulate vascular environment	TNF-*α* quantification, gene expression, protein analysis, and pathway inhibition assays	Apo A-IV upregulated TNF-*α* expression and induced proinflammatory phenotype via NR4A3, supporting its role in vascular inflammation in DFUs
[33]	Human pancreatic, liver, and vascular cells in a multiorgan chip	Emulate pathophysiological features of T2DM using 3D-printed OoC	Integration of islet, liver, and vascular compartments under perfused conditions to model systemic T2DM	Vascular endothelial layers printed using bioinks in vascular chamber of chip	Long-term co-culture (2+ weeks) with dynamic perfusion mimicking metabolic interactions	Metabolic assays, insulin/glucose levels, angiogenic response, cytokine profiling, histology	Demonstrated insulin resistance, hyperglycemia, endothelial dysfunction and impaired angiogenesis under diabetic conditions
[31]	FBs from 03 DFUs and non-diabetic control samples	Present a new 3D skin disease model for evaluating the function of macrophages in individuals with DM	Two types of experiments were conducted: one assessing the behavior of macrophages incorporated into HSEs, and the other examining the impact of patient-specific monocytes on HSEs	The construction of HSEs in triplicate wells. HSEs were created by mixing NFFs or DFUFs with macrophages and Type I Collagen. After collagen remodeling by fibroblasts, keratinocytes were added to the surface	FBs with or without macrophages added to collagen. Neonatal keratinocytes were seeded on top for differentiation. The tissue was cultured at air-liquid interface	Histological analysis, immunofluorescence, gene expression and inflammatory cytokines	Recapitulated diabetic inflammatory phenotype using monocytes and fibroblasts from diabetic patients
[34]	Monocytes and fibroblasts from DFU patients	Develop a physiologically relevant 3D human skin model to study macrophage function in diabetic wound healing	Incorporated DFU-derived fibroblasts and blood monocytes into HSEs to observe macrophage differentiation and inflammatory response	HSEs formed with fibroblasts and monocytes embedded in collagen matrix, overlaid with keratinocytes, and cultured at air-liquid interface	Fibroblasts and monocytes cocultured; keratinocytes added for stratification; tissues differentiated over 1 week	Histology, immunofluorescence, cytokine quantification (IL-1*β*, IL-6, IL-8), ELISA, gene expression	Diabetic macrophages displayed M1 phenotype, with increased proinflammatory cytokine secretion; fibroblasts influenced macrophage polarization, validating the model for DFU inflammation and drug testing
[35]	Human fibroblasts exposed to high glucose	Evaluate the effect of hyperglycemia on wound healing using in vitro 3D models	Exposed fibroblasts to high glucose and assessed cellular behavior in ECM-like environment	Fibroblasts embedded in collagen matrix forming 3D dermal equivalents	High glucose conditions simulated diabetic environment	Morphology, collagen contraction, viability, inflammatory markers (IL-6, MMPs)	High glucose impaired fibroblast function, reduced contraction, and increased inflammatory markers, simulating diabetic wound environment
[23]	HUVECs from diabetic patients, diagnosed for at least 4 years	Develop a 3D model of diabetic skin to study wound healing pathophysiology	HUVECs were cultured on 3D Matrigen platform. Effects of compound GK-2 were evaluated for healing	Cells expanded in culture plates, transferred to 3D platform, then incubated under controlled conditions	24 mm transparent wells, 15 mm deep, incubated at 37°C with 5% CO_2_	Cell viability, proliferation, histology, immunofluorescence, gene expression, hydroxyproline, fibronectin, MMPs	The model mimicked diabetic skin features. GK-2 showed significant improvements in wound healing parameters
[17]	FBs originating from DFUs	Develop a 3D tissue model to mimic characteristics of chronic diabetic wounds	Isolated fibroblasts from DFUs, grew them in collagen-fibrin hydrogels, and characterized tissue	Cell culture derived from biopsied fibroblasts; seeded in collagen/fibrin matrix	Not specified	Histology, immunofluorescence, gene expression, viability, matrix contraction	Successfully reproduced DFU characteristics including ECM remodeling, keratinocyte behavior, and impaired healing

**Legend:** DM = Diabetes Mellitus; HUVECs = Human Umbilical Vascular Endothelial Cells; DHSM = Type 2 Diabetic Human Skin Model; HSEs = Human Skin Equivalents; ECM = Extracellular Matrix; MPP = Matrix Metalloproteinase; FBs = Fibroblasts; DFU = Diabetic Foot Ulcer; NFFs = Normal Skin Fibroblasts; DFUFs = Diabetic Foot Ulcer Fibroblasts.

[23] developed a 3D diabetic skin model by isolating human umbilical vein endothelial cells, dermal fibroblasts, and keratinocytes from type 2 diabetic patients diagnosed for at least four years. The cells were cultured on a 3D GelMA cell culture platform crosslinked at 8%, with a compression modulus of 4.53 ± 0.67 kPa. For the construction of in vitro skin models, hydrogels like GelMA are commonly used due to their high water content, high permeability to small molecules, mechanical biocompatibility, and adjustable physicochemical properties that mimic those of the skin. In the methodological development, [23] used primary skin cells from diabetic patients but encountered difficulties in obtaining sufficient material for all assays. Therefore, they performed cell culture studies in the hydrogel using normoglycemic human dermal fibroblasts for optimization. Then, an in vitro 3D diabetic skin model was constructed using keratinocytes, dermal fibroblasts, and HUVECs derived from type 2 diabetic donors. The in vitro 3D diabetic skin model was then characterized and compared with the in vivo diabetic skin, showing significant similarities.

According to [23], the skin model featured a dermal layer and a continuous basal epidermal layer, which exhibited a structure akin to a blood capillary, measuring 12 mm in diameter and 1.86 mm in height. The cells were cultivated in a supplemented medium containing human blood plasma to replicate the natural cellular environment. To simulate the blood composition of individuals with diabetes, the glucose concentration in the medium was elevated to around 25 mM. The medium was renewed three times per week. For wound healing analysis, a biopsy was conducted, creating a 4-mm synthetic wound at the center of the diabetic skin model, with a sample size of n = 3. The solution consisted of atelocollagen at 6.67 mg/mL, L-ascorbic acid at 6.67 mg/mL, and sodium alginate at 1.67 mg/mL. Subsequently, the samples underwent incubation, cellular fixation, and subsequent analysis.

To cultivate the dermis, human umbilical vein endothelial cells (HUVECs) were isolated via enzymatic digestion. The presence of endothelial cells was confirmed by the expression of the CD31 marker, indicating their presence. Epithelial cells, specifically keratinocytes, were immunolabeled using cytokeratin 5 to ensure a pure culture. Under microscopic analysis, structures resembling capillaries were observed beneath the epidermal layer, although the vascularization of the tissue could not be assessed. [23] hypothesized that this limitation is attributable to the lack of perfusion in the culture, which resulted from the substantial thickness of the hydrogel used for cultivation. This may have prompted cell migration toward the outer regions and hindered the appropriate interaction between fibroblasts and endothelial cells. These findings demonstrate that the 3D diabetic skin prototype could identify inflammation in diabetic skin samples in a laboratory setting, mirroring in vivo conditions. This implies that the prototype has the potential to investigate inflammation in diabetes, a significant factor in the development of the disease. Moreover, an in vitro 3D diabetic skin model was employed to assess the effectiveness of topical therapies for diabetes. The GelMA hydrogel was crosslinked at a concentration of 8%. The authors noted that, unlike the results of previous studies, this concentration adequately facilitated the dispersion and normal functioning of dermal fibroblasts in 3D cultures.

After 9 days of cultivation, the curative substance exhibited the migration of keratinocytes from the periphery to the core of the injury, albeit failing to encompass the area entirely. Using qualitative assessment, which involves observing typical cellular morphology, and quantitative evaluation, encompassing cell migration and cell enumeration, the therapeutic hydrogel revealed beneficial outcomes that hold potential for improvement in diverse frameworks aimed at addressing the recovery of persistent wounds [23].

The study by [34] presents an innovative three-dimensional human skin equivalent (HSE) model that incorporates fibroblasts derived from patients with diabetic foot ulcers (DFUs) and blood monocytes, which differentiate in situ into macrophages. This model accurately simulates chronic inflammatory aspects observed in DFUs, demonstrating that macrophages derived from diabetic patients exhibit a characteristic pro-inflammatory M1 profile, with increased secretion of cytokines such as IL-1*β*, IL-6, and IL-8. Moreover, the results demonstrate that diabetic fibroblasts directly influence macrophage polarization, underscoring the significance of cell-cell interactions within the wound microenvironment. This model represents a significant advancement in the study of inflammation in DFUs and shows promise as a platform for testing regenerative and anti-inflammatory therapies in a more physiologically relevant context than traditional two-dimensional models [34].

[17] concluded that 3D models as a platform for the growth and analysis of DFUs from a biological perspective are the way to map the critical aspects related to highlighted mechanisms for cell-cell and cell-matrix interactions. The authors selected fibroblasts derived from DFU patients and control patients with compatible regulation and DFU formation in their investigation. The selection of the 3D structure is superior to 2D culture models, as the latter fails to provide the necessary complexity of inquiry of the process of angiogenesis and interactions between fibroblasts and keratinocytes in the formation of the extracellular matrix (ECM). The studied model accurately reproduced the main stages, including reduced angiogenesis stimulus, increased proliferation of keratinocytes, decreased reepithelialization, and impaired ECM deposition.

[17] stimulated ECM production by introducing ascorbic acid into a 3D culture for 5 weeks. As a result, granulation tissue similar to extracellular matrix (ECM) was observed, with the experimental group producing a thinner structure than the control group. These findings suggest that the phenotype of pressure ulcers, in terms of ECM production, may contribute to the chronic nature of wounds.

To characterize the healing potential of isolated fibroblasts, [17] conducted in vivo experiments on male mice 16 weeks old, in which two layers of skin measuring 6 mm were obtained from the dorsal area of each animal. The fibroblasts encapsulated in the hydrogel were tested for their healing potential. This allowed the study of how the encapsulated cells behaved and interacted with the wound-healing environment, providing valuable information about the therapeutic potential of the hydrogel in tissue regeneration using animal models. The 400 groups cultivated from DFU-derived cells showed reduced cell motility during the wound repair process.

The hydrogel used alginate with a high content of G blocks, having a mannuronic-guluronic acid (MVG) ratio of M: G = 40:60, with a molecular weight of approximately 250 kDa. The gels contained a concentration of 2% alginate, with a high molecular weight to low molecular weight ratio of 25:75. These gels were ionically crosslinked with a 1.22 M calcium sulfate solution, which served as the cell culture environment. To analyze the crosstalk between fibroblasts and keratinocytes in the 3D environment, human skin equivalents (HSEs) were constructed in triplicate wells at a final concentration of 3 × 10^5^ cells/mL. They were submerged for one week in an epidermal collagen growth medium containing 3.8 g/L glucose and 0.3% serum [17].

To evaluate the hyperproliferative characteristics of keratinocytes in DFUs in the 3D model, tissues from diabetic wounds and nonulcerated skin were included in collagen matrices of the dermal compartment of the HSE. A biopsy was performed in the center of the tissue to create a wound. The base of the injured tissue was the same for the experimental and control groups, constructed from fibroblasts and keratinocytes derived from a healthy human foreskin. In the structures tested with DFU-derived fibroblasts, basal keratinocytes in the HSE showed 44%–47% higher proliferation rates, consistent with findings in the literature. Regarding wound closure, gels filled with DFU-derived cells achieved an average reepithelialization rate of 36% [17].

Regarding angiogenesis, cells derived from DFUs showed reduced endothelial cell sprouting compared to the control group, with lower secretions of IL-6, IL-8, and SDF-1. [17] suggest that the model they developed, incorporating endothelial cells in gels, can be used to study the crosstalk relationship between endothelium and fibroblasts. This results in more precise studies on the influence of angiogenesis disorders.

Analysis of ECM disorders in 3D models is vital for understanding wound healing. The production of ECM fibroblasts plays an essential role, serving as a support for keratinocyte migration. The hydrogel used in this study comprised hydrophilic polymers that provided a 3D environment for cell proliferation and interaction. The outcomes presented in both of the reviewed studies demonstrated the potential application of hydrogels for structuring the tissue layers of chronic wounds [17,23]. In the following section, we discuss the evidence of these applications in microfluidics studies, bringing future perspectives for application in the context of DFUs.

A study conducted by [31] aimed to incorporate macrophages from diabetic patients into a 3D human skin model. In this study, three groups of primary dermal fibroblasts were isolated from patient biopsies: fibroblasts derived from type 2 diabetic foot ulcers (DFUFs), fibroblasts derived from type 2 diabetic, nonulcerated feet (DFFs), and fibroblasts derived from non-diabetic, nonulcerated feet (NFFs), totaling 12 fibroblast strains (4 in each group). Bright-field microscopy revealed that the isolated cells had a spindle-like morphology, which is a typical characteristic of dermal fibroblasts [31].

To ensure the purity of the fibroblasts, considering the heterogeneous cellular composition of the dermis, a flow cytometry analysis was performed to identify mesenchymal markers expressed by skin fibroblasts, including CD73, CD105, and CD140b. A well-characterized human foreskin fibroblast line (HFF) was used as a positive control for comparison. All patient-derived fibroblasts demonstrated high expression of mesenchymal markers, with over 99% positive for CD73, over 95% positive for CD105, and over 92% positive for CD140b, which was similar to the control HFF fibroblasts (95%, 95%, 99% positive, respectively [31].

To rule out the presence of contaminating cell types, such as endothelial cells, macrophages, and lymphocytes, the expression of the hematopoietic marker CD31 was also assessed. All fibroblast cultures demonstrated low expression of the hematopoietic marker, with fewer than 3% of cells positive for CD31, a result similar to that of the HFF control (2% positive). There were no significant differences in the expression of markers between the fibroblast groups. These data indicate that the cells isolated from patient biopsies were predominantly fibroblastic, with minimal presence of contaminating cell types [31].

The study by [12] provides relevant evidence by demonstrating, through a microfluidic arterial model, that apolipoprotein A-IV (Apo A-IV) isolated from patients with diabetic foot ulcers (DFUs) induces a significant inflammatory response in human endothelial cells. Under dynamic flow conditions, typical of OoC devices, the authors observed increased expression of tumor necrosis factor-alpha (TNF-*α*), mediated by the NR4A3 signaling pathway. These findings reinforce the active role of systemic and vascular alterations in the pathophysiology of diabetic foot ulcers (DFUs), highlighting Apo A-IV as a potential therapeutic target to modulate chronic inflammation and endothelial dysfunction in diabetic wounds. Furthermore, the use of a microfluidic platform enabled a more accurate simulation of the human vascular microenvironment, representing a substantial advancement over conventional models [12].

The DFUFs and DFFs induced less endothelial cell sprouting than did the NFFs in a 3D angiogenesis model. Cytokine analysis revealed that the DFUFs secreted smaller amounts of IL-6, IL-8, and SDF-1 than the NFFs. Since these factors play a significant role in angiogenesis, and their production is altered in diabetic ulcers, the hypothesis that the modified cytokine secretion by DFUFs and DFFs would lead to reduced angiogenic induction in 3D models was tested by conducting a 3D in vitro angiogenesis assay. It was observed that NFF10 stimulated endothelial cell sprouting to a greater extent than DFUF1 (3.7 times more) and DFF6 (1.6 times more). These results suggest that this model can be utilized to investigate the communication between endothelial cells and fibroblasts derived from diabetic ulcers, as well as how this interaction may contribute to impaired angiogenesis in chronic wounds [31].

A recent study by [35] developed a pancreas-liver organ-on-chip coculture model that effectively recapitulates the physiological crosstalk between pancreatic islets and hepatocytes relevant to type 2 diabetes mellitus (T2DM). The microfluidic platform, utilizing rat-derived primary hepatocytes and islets of Langerhans, demonstrated that insulin secreted by the islets was sufficient to restore liver-specific functions, such as albumin synthesis and the expression of metabolic markers (e.g., CYP3A2, GLUT2). The coculture not only preserved hepatic morphology and function in the absence of exogenous insulin but also enhanced insulin and C-peptide secretion from the islets. Gene expression analyses confirmed the bidirectional regulation between tissues, supporting the platform’s potential for modeling glucose-insulin dynamics and liver-pancreas interactions. This organ-on-chip model offers a valuable in vitro alternative for investigating diabetic pathophysiology and screening antidiabetic therapies without the use of animal models [35].

### Quality and risk of bias in individual studies

The risk of bias assessment was carried out using criteria adapted from the Generic ROB tool. With the inclusion of new studies in this review, the methodological landscape has slightly broadened, although some important gaps remain. Among the seven studies included, most provided a clear description of tissue and cell selection criteria, as well as the presence of experimental and control groups. However, critical aspects such as random allocation of cells on 3D platforms, blinding of outcome assessors, reporting of losses during assays, standardized data collection, and control of confounding factors were generally underreported or missing.

Two studies stood out for presenting a low overall risk of bias [23,31], demonstrating stronger methodological rigor. Nevertheless, despite the identified limitations, the information gathered remains relevant and contributes meaningfully to the current state of knowledge in this field. It is worth emphasizing that this is still an emerging area, where 3D culture technologies and microfluidic platforms for diabetic foot ulcers (DFUs) are under development. As research progresses, methodological standards and quality criteria are expected to improve.

**Fig 3:**
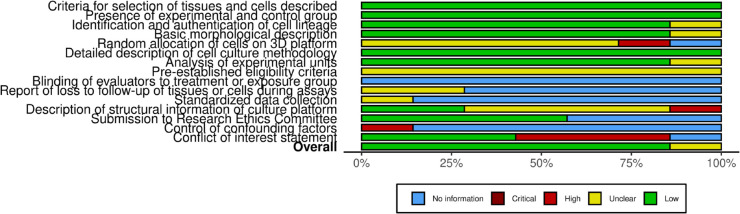
Overall quality of the selected studies. %“LOW" - Low risk of bias. “NO INFORMATION" - Insufficient information to assess the risk of bias. “CRITICAL" - High risk of bias, with significant limitations. “HIGH" - High risk of bias, with substantial limitations. “UNCLEAR" - Insufficient available information. A detailed description of the evaluated parameters is found in Supplementary material.

Fig 3 provides a summary of the risk of bias assessment for the included studies. More detailed information on the parameters evaluated can be found in the supplementary material.

## Discussion

### Advances in organ-on-a-chip vascularization

Our systematic review aimed to explore the advancements in 3D cell culture technologies as applied to diabetic foot ulcers (DFUs), with a particular focus on organ-on-a-chip (OoC) platforms. From the comprehensive literature screening, only three studies met the inclusion criteria. These selected works did not present fully developed DFU models within OoC systems. Nonetheless, they employed 3D culture strategies, including hydrogel-based platforms, to replicate dermal and epidermal structures. Despite mimicking in vivo skin architecture, these models still fall short in replicating the intricate cellular and molecular interactions that occur in human skin. Based on evidence from other pathological contexts, we further discuss the potential for integrating such approaches into microfluidic systems.

Understanding the complex pathophysiological mechanisms underlying the onset of DFU and impaired healing remains crucial. Key contributing factors include neuropathy, ischemia, infection, prolonged hyperglycemia, persistent inflammation, and alterations in the extracellular matrix (ECM) [4]. Approximately half of DFU cases involve vascular complications, often associated with peripheral arterial disease (PAD), predominantly atherosclerosis. This condition is marked by endothelial injury, vascular smooth muscle dysfunction, hypercoagulability, platelet abnormalities, and inflammatory responses [4].

Angiogenesis is tightly regulated by a balance between pro-angiogenic factors (e.g., TGF-*β*, TNF-*α*, VEGF, PDGF, FGF) and anti-angiogenic mediators (e.g., angiostatin, TIMP-2, TSP-1, endostatin), alongside oxidative stress markers like HIF-1*α* and inflammatory pathways such as Sonic Hedgehog signaling. While increased angiogenesis is critical in the early phases of wound healing, its downregulation is essential for scarless tissue repair. Diabetes disrupts this balance, altering the expression of angiogenic mediators due to hyperglycemia, necrosis, gangrene, tissue fibrosis, thrombosis, and differential cellular responses across ulcer types. These variations also complicate the translational relevance of findings from animal models to human clinical applications [36].

This review highlights the progress and limitations of 3D culture systems in modeling DFUs. Although the included studies did not directly apply OoC technologies, they successfully developed stratified dermal structures. They highlighted the challenges of replicating the complexity of diabetic human skin in vitro [17,23].

[37] highlights the expanding role of microfluidic technologies in diabetes management, bridging the gap between in vitro modeling and clinical applications. The review discusses innovative non-invasive diagnostics—such as glucose sensors using alternative body fluids and devices for HbA1c and insulin detection—illustrating how these systems may address limitations of conventional, often painful, methods. Additionally, advances in transdermal, oral, intraperitoneal, and inhalable drug delivery approaches reinforce the therapeutic potential of microfluidic devices, which remains mainly in the preclinical stages. These innovations are particularly relevant to DFUs, where sustained glycemic control is vital for both prevention and treatment. The integration of OoC platforms, biosensors, and drug delivery technologies supports a vision of personalized diabetes management through a synergistic technological ecosystem.

In a complementary study, [38] employed 3D bioprinting to construct vascularized, multilayered human skin equivalents using keratinocytes, fibroblasts, and endothelial cells. These constructs simulate type 2 diabetic conditions—chronic hyperglycemia, persistent inflammation, and angiogenic dysfunction—producing clinical-like alterations such as aberrant ECM deposition and reduced vascular density. The model offers a human-relevant platform for testing regenerative therapies and pharmacological agents, providing a controlled and reproducible simulation of diabetic skin microenvironments.

Furthermore, [39] emphasizes the importance of integrating immunocompetent components into DFU-on-a-chip systems. Chronic inflammation, a hallmark of DFU pathology, is inadequately modeled by traditional in vivo or 2D systems. The authors advocate for incorporating diseased human cells, such as macrophages, keratinocytes, and fibroblasts, into scaffolds that facilitate pathological extracellular matrix (ECM) deposition. These constructs, when coupled with microfluidic platforms, more accurately replicate the pro-inflammatory stagnation observed in non-healing diabetic wounds. Their insights expand our understanding of critical features for robust preclinical DFU models, including innate immune responses and dysregulated dermal microenvironments.

### Organs-on-a-chip

The advent of OoC technology has marked a transformative era in biomedical research. By enabling high-fidelity simulation of human physiology and disease, this approach addresses the longstanding limitations of conventional in vitro systems and animal models [40]. Coined in 2010 by researchers at Harvard’s Wyss Institute, the concept emerged from pioneering work in the late 1990s and early 2000s. Since then, it has gained momentum, evolving into a cornerstone of translational research [41–43].

Microfluidic models facilitate the study of vascular responses to mechanical and biochemical stimuli. The endothelium, a central regulator of vascular homeostasis, functions as a selective barrier and modulator of hemostasis and angiogenesis [44]. In vitro platforms allow analysis of endothelial behavior in diabetes-related vascular dysfunction [45].

3D skin models provide insights into keratinocyte-fibroblast interactions and extracellular matrix (ECM) dynamics. These include scaffold-free, self-assembled skin and scaffold-based human skin equivalents (HSE), both of which are used in pharmaceutical and cosmetic testing. The methodologies developed for these systems provide a foundation for adapting similar approaches to DFU modeling [20].

**Fig 4:**
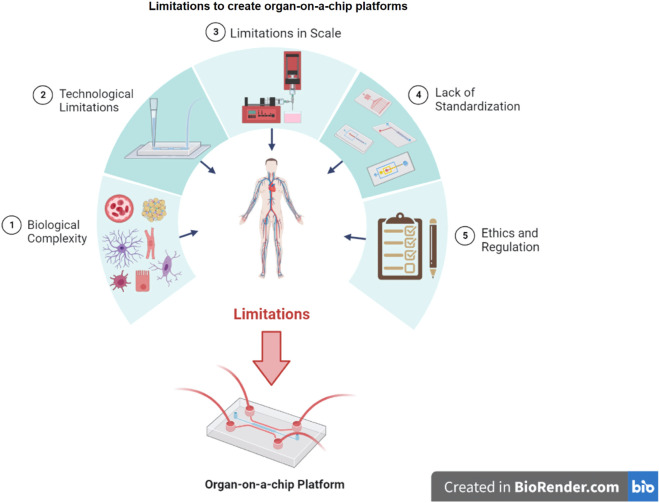
Limitations to create organ-on-a-chip platforms: (1) Biological complexity: Difficulty in reproducing cellular interactions due to the complexity that encompasses the microenvironment, metabolic activity, presence of blood vessels, and interaction with the immune system. (2) Technological limitations: The technologies used to create organ-on-a-chip platforms are still under development and have some limitations, such as the lack of miniaturization and standardization of some components, the difficulty in creating ideal culture conditions for all cells and tissues involved, and the limited detection of molecules at low concentrations. (3) Scale limitations: Mass production of *organ-on-a-chip* platforms is still complex, making these platforms expensive and limited to small-scale studies. Their use for large-scale drug testing still needs to be improved. (4) Lack of standardization: The lack of standardization in fabricating and using *organ-on-a-chip* platforms hinders the comparison and replication of results among different studies and institutions. (5) Ethics and regulation: Ethical and regulatory issues are involved in the use of these platforms, such as the use of human and animal tissues, the lack of specific regulation for this type of technology, and the need to ensure the safety and efficacy of drugs tested on humans.

Microfluidic angiogenesis assays investigate endothelial sprouting, migration, and vessel maturation using endothelial monolayers or channels molded in materials such as polydimethylsiloxane [44]. Despite these advancements, challenges remain, such as reliance on non-primary cell sources and exogenous factor stimulation, which may limit pathological relevance [1]. Moreover, technical complexity in chip operation, including pump calibration and media control, presents logistical barriers to widespread adoption [45] (Fig 4).

### Specificities of 3D cell culture in diabetic foot ulcers

The current emphasis has been on constructing a 3D rendition of DFUs. Researchers have successfully fabricated a 3D model of a diabetic wound, employing fibroblasts and keratinocytes obtained from individuals diagnosed with type 2 diabetes. By culturing the model in a medium supplemented with glucose, the model accurately reproduces the hyperglycemic microenvironment characteristic of DFUs. However, the lack of a normoglycemic model hampers the evaluation of treatment efficacy through comparison. Although the process of dermal vascularization remains unobservable, employing a denser hydrogel with enhanced medium perfusion could potentially improve visual clarity [23].

The heterogeneity of fibroblasts and the microenvironmental niche within the dermis also play crucial roles in wound healing. Fibroblasts derived from the papillary dermis show better healing capacity than those derived from the reticular dermis, while fibroblasts from the palmoplantar regions show reduced capacity. Additionally, the cell origin and local microenvironment influence the response to wound healing [17].

Primary cells retain a “metabolic memory” of the DFU microenvironment even after expansion in culture, highlighting the importance of incorporating cells derived from DFUs into 3D models of hyperglycemic wounds. Researchers have successfully developed biomimetic 3D models of DFUs using fibroblasts derived from patients with DFUs and normal human keratinocytes [17]. However, the use of collagen from animal extracellular matrices introduces variability. To address this issue, [31]. Established a self-organized skin-substitute HSE model that supports the production of the human ECM. By incorporating fibroblasts derived from DFUs, human keratinocytes, and monocytes, the SASS model offers greater consistency and scalability for screening. Incorporating monocytes derived from diabetic patients resulted in a pro-inflammatory condition, mimicking the M1 macrophage phenotype observed in diabetic foot ulcers (DFUs). These advances provide a more accurate representation of DFUs and offer potential endpoints for therapeutic development. The limited availability of biopsy samples and the potentially small growth of DFU cells pose a challenge [17,31].

Regarding the commercialization of in vitro 3D skin models, products like EpiSkin^™^, T-Skin^™^ (L’Oréal, Paris, France), Epiderm^™^, and EpiDermFT^™^ (MatTek, Ashland, MA, USA) have been used as full-thickness or epidermal models for drug administration analysis, sensitization, and wound healing. However, they still fall short of faithfully reproducing the physiological characteristics of human skin. One of the main shortcomings of these skin models is that they do not recapitulate the active transport of molecules such as nutrients, growth factors, and specific cell interactions [17,20].

Numerous methodologies exist for fabricating 3D skin models in a laboratory setting, including the use of hydrogels as supportive structures for the dermis while culturing keratinocytes on top of them. Nevertheless, these models cannot faithfully replicate the intricate molecular transport and migration of cells observed in vivo, primarily due to the lack of a functional capillary network system [20]. Additionally, these models predominantly employ normoglycemic cells, and there is a scarcity of available models utilizing primary cells derived from patients with type 2 diabetes. Hydrogels, particularly gelatin-based ones, have been widely used due to their modifiable characteristics, permeability, and biocompatibility. However, despite significant progress, inherent constraints persist in the construction of in vitro 3D skin models that accurately recapitulate the physiology of human skin. Another impediment pertains to the inability to fabricate in vitro 3D skin models with a thickness exceeding 100 2000 μm [17,23].

### Advances in organ-on-a-chip vascularization

From the comprehensive literature screening, only three studies initially met the inclusion criteria. With the inclusion of four additional studies following a refined search strategy, the review now encompasses seven studies. These selected works did not present fully developed DFU models within OoC systems. Nonetheless, they employed 3D culture strategies, including hydrogel-based platforms to replicate dermal and epidermal structures. Despite mimicking in vivo skin architecture, these models still fall short in replicating the intricate cellular and molecular interactions of human skin. Based on evidence from other pathological contexts, we further discuss the potential for integrating such approaches into microfluidic systems.

Understanding the complex pathophysiological mechanisms underlying DFU onset and impaired healing remains crucial. Key contributing factors include neuropathy, ischemia, infection, prolonged hyperglycemia, persistent inflammation, and extracellular matrix (ECM) alterations [4]. Approximately half of DFU cases involve vascular complications, often associated with peripheral arterial disease (PAD), predominantly atherosclerosis. This condition is marked by endothelial injury, vascular smooth muscle dysfunction, hypercoagulability, platelet abnormalities, and inflammatory responses [4].

Angiogenesis is tightly regulated by a balance between pro-angiogenic factors (e.g., TGF-*β*, TNF-*α*, VEGF, PDGF, FGF) and anti-angiogenic mediators (e.g., angiostatin, TIMP-2, TSP-1, endostatin), alongside oxidative stress markers like HIF-1*α* and inflammatory pathways such as Sonic Hedgehog signaling. While increased angiogenesis is critical in the early phases of wound healing, its downregulation is essential for scarless tissue repair. Diabetes disrupts this balance, altering the expression of angiogenic mediators due to hyperglycemia, necrosis, gangrene, tissue fibrosis, thrombosis, and differential cellular responses across ulcer types. These variations also complicate the translational relevance of findings from animal models to human clinical applications [36].

The present review underscores the progress and limitations of 3D culture systems in modeling DFUs. Though the included studies did not directly apply OoC technologies, they succeeded in developing stratified dermal structures and highlighted the challenges of replicating the complexity of diabetic human skin in vitro [17,23].

[37] underscores the growing importance of microfluidic technologies in integrated diabetes care, expanding the discussion toward clinically relevant applications. The authors review novel non-invasive diagnostic tools—such as glucose sensors using alternative body fluids and HbA1c/insulin detection devices—that may overcome pain and adherence limitations of traditional approaches. Furthermore, by exploring drug delivery via transdermal, oral, intraperitoneal, and inhalation routes, the study demonstrates the versatility of microfluidic structures for therapeutic use, despite remaining largely in preclinical stages. These insights are particularly valuable for DFU care, as glycemic control remains pivotal to both prevention and treatment. Ultimately, the study highlights the potential for integrated ecosystems in which OoC models, biosensors, and drug-delivery systems converge for personalized diabetes management.

[38] utilized 3D bioprinting to create multilayered, vascularized human skin equivalents composed of keratinocytes, fibroblasts, and endothelial cells. This model recapitulates features of type 2 diabetes—including chronic hyperglycemia, persistent inflammation, and impaired angiogenesis—leading to aberrant ECM deposition, reduced microvascular density, and altered cytokine expression. The platform represents a significant advance in diabetic wound modeling and offers a humanized system to evaluate regenerative therapies and biomaterials under controlled conditions.

[39] emphasized the importance of incorporating immunocompetent elements into DFU-on-a-chip systems. Chronic inflammation, a key contributor to non-healing wounds, is inadequately modeled by standard in vivo or 2D platforms. The study proposes integrating diseased human cells—including macrophages, fibroblasts, and keratinocytes—into scaffolds that replicate pathologic ECM deposition. Coupled with microfluidic systems, these models can emulate the inflammatory stagnation seen in DFUs, reinforcing the necessity of immune-responsiveness and microenvironmental fidelity in robust preclinical platforms.

[46] introduced a co-culture microphysiological platform linking human pancreatic islets and liver spheroids. This model reveals the dynamics of insulin secretion and hepatic glucose uptake, central to understanding insulin resistance in diabetic patients. [47] developed an innovative WAT-on-a-chip platform, integrating mature human white adipocytes into a perfused microfluidic system. The model preserved cell viability and function for over 30 days, enabling real-time monitoring of lipid metabolism and pharmacological response—highly relevant for addressing chronic inflammation and impaired healing in obese, diabetic patients. Though not explicitly developed for DFUs, both models offer promising frameworks to integrate into future DFU-specific platforms, reflecting the systemic nature of wound pathophysiology.

[24] engineered a hydrogel-based blood vessel-on-a-chip, forming vascular channels lined with endothelial cells and simulating blood flow via microfluidics. The system enabled analysis of endothelial functions—barrier integrity, inflammatory responses, and cell-cell interactions—and demonstrated excellent channel morphology and cell viability, supporting its potential for structured vascularization in OoC platforms.

[48] developed an injectable PEG-gelatin hydrogel that supported adipose-derived stem cell (hASC) viability and metabolic activity for extended periods. In diabetic mouse wound models, the hydrogel demonstrated biocompatibility, reduced inflammation, promoted angiogenesis, and accelerated healing, validating its application in chronic diabetic wounds.

Though not directly evaluated in the included studies, [49] highlighted the relevance of temperature-sensitive hydrogel valves in microfluidic platforms. These structures enabled dynamic flow control, critical for maintaining optimal environmental conditions during in vitro culture.

Altogether, these findings emphasize the need for integrated, immune-competent, and metabolically dynamic platforms to more accurately represent DFU pathophysiology in vitro. Future research should prioritize the combination of hydrogel scaffolds, microfluidics, 3D bioprinting, and systemic comorbidity modeling to develop translationally robust platforms for therapeutic testing and disease understanding.

## Limitations

The primary limitations of this study were the variability in the nomenclature of 3D cell culture, which initially made it difficult to differentiate between structures that were devices with microfluidics or that provided 3D culture structures; the limited availability of studies related explicitly to DFUS due to the challenges in obtaining tissue samples from these patients; the low number of studies on the topic, which impacted the qualitative and quantitative analyses of the review; the presentation of results focused on qualitative data, where some findings may be influenced by the evaluator involved in the study; and the no nutilization of other biomaterials or structures associated with microfluidic devices that could have enhanced the results obtained.

## Conclusion

Biomedical and translational research in health is evolving rapidly, particularly with the advancement of OoC devices, which offer innovative avenues for more accurate, ethical, and clinically relevant experimentation. These platforms aim to bridge the existing gaps between conventional in vitro models, animal testing, and clinical applications.

Diabetes is a chronic condition with high morbidity and mortality, presents significant challenges for healthcare systems globally. Among its complications, diabetic feet are particularly burdensome, with limited evidence supporting effective prognostic and therapeutic strategies. Our systematic review focused on current 3D cell culture techniques used to model DFUs, specifically in microcontroller environments that simulate human physiology more closely.

Of the seven studies included in this review, all employed hydrogel-based structures to replicate dermal and epidermal tissues using cells from diabetic patients or ulcer biopsies. These models successfully demonstrated the hallmark features of diabetic skin, including impaired wound healing and altered cellular responses in a hyperglycemic environment. However, none of the models incorporated fully structured vascular elements, underscoring a critical limitation in reproducing chronic wound pathophysiology in vitro.

Future developments must address several interconnected challenges. First, greater access to patient-derived tissues, including biopsies and debrided material, is necessary to broaden the range and fidelity of cell cultures. Second, microfluidic chip designs require refinement to more effectively emulate layered and vascularized skin architecture. Improvements in biomaterials and 3D printing methods are also vital, particularly in terms of producing permeable, stable, and structurally precise platforms suitable for drug testing and physiological modeling. Moreover, there is an urgent need to integrate omics-based approaches, such as metabolomics and proteomics, which can provide a deeper understanding of wound healing dynamics; however, their implementation remains limited by cost and technical complexity.

Although angiogenesis-related biomarkers were identified, the absence of structured vascular components in the reviewed studies highlights an area for significant progress. The combination of bioprinting, advanced hydrogels, and integration of multi-tissue systems with immune and metabolic responsiveness offers a promising path forward. These innovations could enhance the translational value of in vitro DFU models, enabling more personalized and predictive therapeutic strategies.

In conclusion, this review highlights the importance of multidisciplinary collaboration among tissue engineering, microfluidics, and clinical research to overcome current limitations and accelerate the development of more representative DFUs models. Such efforts are essential to advancing wound care for diabetic patients and improving outcomes in real-world settings.

## Supporting information

S1 Fig Graphical abstract. (TIFF)

S1 File String. Description of the string used in the study. (PDF)

S2 File Database search and results. Detailed database search strategy and results. (PDF)

S3 File Online forms. Online forms used for data extraction. (PDF)

S4 File Excluded articles. List of excluded articles and reasons for exclusion. (PDF)

S5 File Risk of bias. Risk of bias assessment for included studies. (PDF)

S6 File Traffic light plot. Traffic light plot summarizing the risk of bias assessment across studies. (PNG)
