# Supplementary figures and images for: Advanced microfluidic and 3D cell culture platforms for modeling vascularization in diabetic foot ulcers: A systematic review of translational challenges and perspectives
Source: PLoS One. 2026 Apr 6;21(4):e0328278. doi: 10.1371/journal.pone.0328278 (PMC13052901; doi:10.1371/journal.pone.0328278)

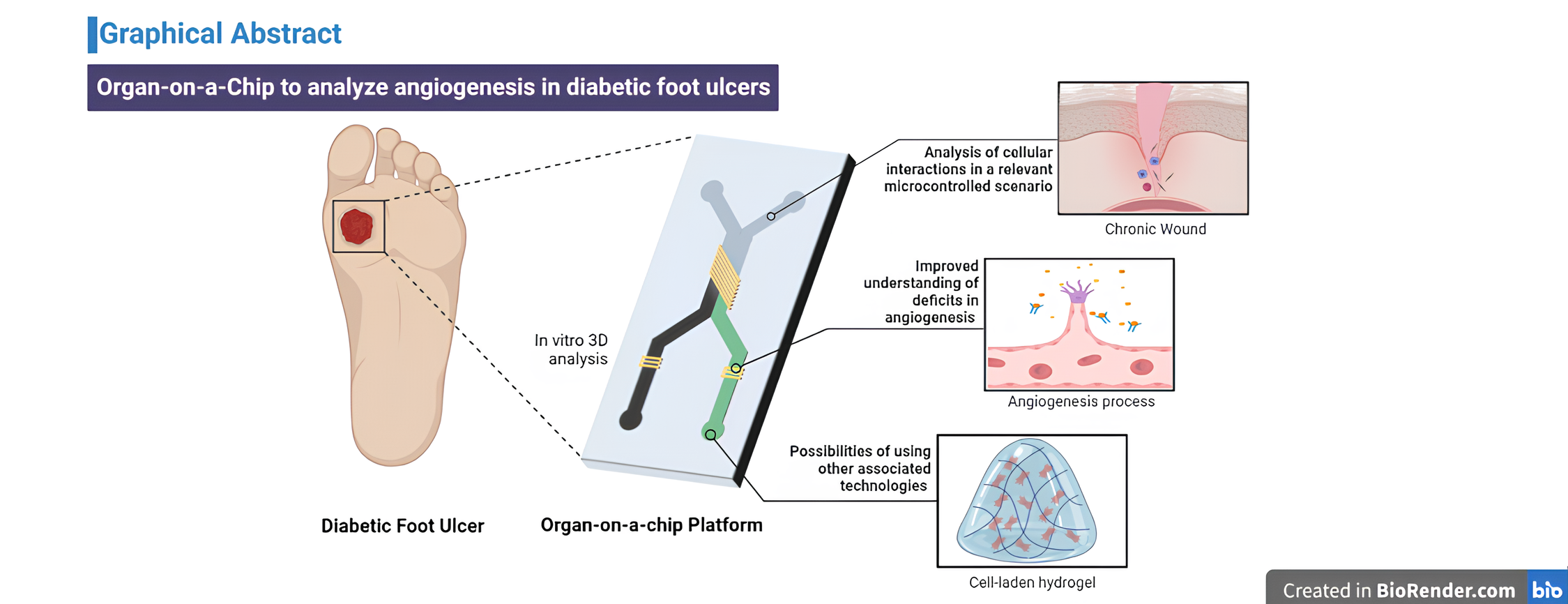

Supplement: S1 Fig — (TIFF) [file pone.0328278.s001.tif]

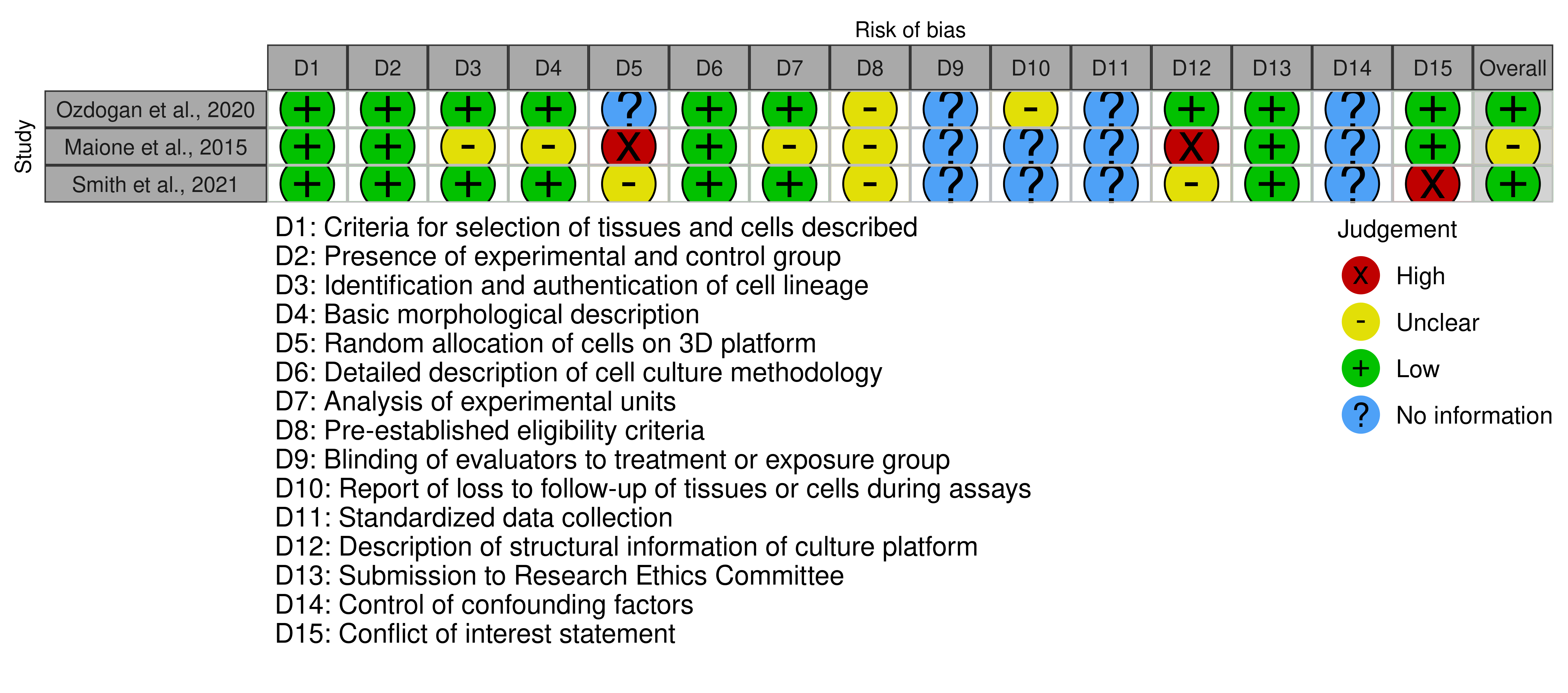

Supplement: S6 File — (PNG) [file pone.0328278.s007.png]
