# Supplementary material for: Advanced microfluidic and 3D cell culture platforms for modeling vascularization in diabetic foot ulcers: A systematic review of translational challenges and perspectives
Source: PLoS One. 2026 Apr 6;21(4):e0328278. doi: 10.1371/journal.pone.0328278 (PMC13052901; doi:10.1371/journal.pone.0328278)
Supplement: S1 File — (PDF) [file pone.0328278.s002.pdf]

Additional File 1 – String

(“Analytical Device*” AND “Microchip”) OR (“Analytical Device*” AND “Nanochip”) OR (“Device*” AND “Lab-On-A-Chip”) OR (“Device*” AND “Microchip Analytical”) OR (“Device*” AND “Microfluidic”) OR (“Device*” AND “Nanochip Analytical”) OR (“Lab On A Chip Devices”) OR (“Lab-On-A-Chip Device”) OR (“Lab-On-A-Chip*” AND “Microfluidic”) OR (“Microchip Analytical Device*”) OR (“Microchip*” AND “Microfluidic”) OR (“Microfluidic Device*”) OR (“Microfluidic Lab On A Chip”) OR (“Microfluidic Lab-On-A-Chip*”) OR (“Microfluidic Microchip*”) OR (“Nanochip Analytical Device*”) OR (“Analytical Device*” AND “Microchip”) OR (“Microchip Analytical Device”) OR (“Analytical Device*” AND “Nanochip”) OR (“In-Check system”) OR (“microfluidic chip”) OR (“body on a chip”) OR Microfluidics OR (“micro□fluidics”) AND (“Agents” AND “Angiogenesis Inducing”) OR (“Agents” AND “Angiogenesis Stimulating”) OR (“Angiogenesis Effect*”) OR (“Angioge* Factor”) OR (“Angiogenesis Inducers”) OR (“Angiogenesis Stimulating Agents”) OR (“Angiogenesis Stimulators”) OR (“Angiogenic Factor” AND “Tumor”) OR (“Effect*” AND “Angiogenesis”) OR (“Factor” AND “Angiogen*”) OR (“Factor” AND “Tumor Angiogenic”) OR (“Inducers” AND “Angiogenesis”) OR (“Inducing Agents” AND “Angiogenesis”) OR (“Stimulating Agents” AND “Angiogenesis”) OR (“Stimulators” AND “Angiogenesis”) OR (“Tumor Angiogenic Factor”) OR (“angiogenesis inducing agent*”) OR (“placental angiogenesis fator”) OR (“Angiogenesis Modulators”) OR (“Modulators” AND “Angiogenesis”) OR (“angiogenesis modulating agent*”) OR (“Angiogenesis” AND “Physiologic*”) OR (“Neovascularization” AND “Physiological”) OR (“Physiologic* Angiogenesis”) OR (“Physiologic* Neovascularization”) AND (“Healing*” AND “Wound”) OR (“Wound Healing*”) OR (“granulation” AND “wound”) OR(“healing” AND “wound”) OR (“repair” AND “wound”) OR (“wound granulation”) OR (“wound regeneration”) OR (“wound repair”) OR Cicatrization OR (Scar*) OR Scarring OR cicatrices OR cicatrix OR (“radiation scar”) AND (“Diabetes Mellitus”) OR (“Complications of Diabetes Mellitus”) OR (“Diabetes Complication”) OR (“Diabetes Mellitus Complication*) OR (“Diabetes Related Complications”) OR (“Diabetes-Related Complication*) OR (“Diabetic Complication*”) OR (“Diabetes Insipidus”) OR (“Diet” AND “Diabetic”) OR (“Prediabetic State”) OR (“Scleredema Adultorum”) OR (“Glycation End Products” AND “Advanced”) OR (“Glucose Intolerance”) OR Gastroparesis OR diabetes OR diabetic OR (“Diabetic Feet”) OR (“Feet” AND “Diabetic”) OR (“Foot Ulcer” AND “Diabetic”) OR (“Foot” AND “Diabetic”) OR (“Diabetic foot”) OR (“Foot” AND “Diabetic”) OR (“Diabetic Feet”) OR (“Feet” AND “Diabetic”) OR (“diabetic feet”) OR (“diabetic foot syndrome”) OR (“diabetic foot ulcer”) OR (“foot ulcer” AND “diabetic”)
