# Supplementary material for: Advanced microfluidic and 3D cell culture platforms for modeling vascularization in diabetic foot ulcers: A systematic review of translational challenges and perspectives
Source: PLoS One. 2026 Apr 6;21(4):e0328278. doi: 10.1371/journal.pone.0328278 (PMC13052901; doi:10.1371/journal.pone.0328278)
Supplement: S2 File — (PDF) [file pone.0328278.s003.pdf]

Additional File 2 – Database search and results

Database	Search	Quantity of Publications
**Scopus**<br>January 24, 2023<br>Update: October 19, 2023	((TITLE-ABS-KEY (("Analytical Device*" AND "Microchip") OR ("Analytical Device*" AND "Nanochip") OR ("Device*" AND "Lab-On-A-Chip") OR ("Device*" AND "Microchip Analytical") OR ("Device*" AND "Microfluidic") OR ("Device*" AND "Nanochip Analytical") OR ("Lab On A Chip Devices") OR ("Lab-On-A-Chip Device") OR ("Lab-On-A-Chip*" AND "Microfluidic") OR ("Microchip Analytical Device*") OR ("Microchip*" AND "Microfluidic") OR ("Microfluidic Device*") OR ("Microfluidic Lab On A Chip") OR ("Microfluidic Lab-On-A-Chip*") OR ("Microfluidic Microchip*") OR ("Nanochip Analytical Device*") OR ("Analytical Device*" AND "Microchip") OR ("Microchip Analytical Device") OR ("Analytical Device*" AND "Nanochip") OR ("In-Check system") OR ("microfluidic chip"))) OR (TITLE-ABS-KEY (("body on a chip"))) OR (TITLE-ABS-KEY (microfluidics OR ("micro-fluidics")))) AND ((TITLE-ABS-KEY (("Agents" AND "Angiogenesis Inducing") OR ("Agents" AND "Angiogenesis Stimulating") OR ("Angiogenesis Effect*") OR ("Angioge* Factor") OR ("Angiogenesis Inducers") OR ("Angiogenesis Stimulating Agents") OR ("Angiogenesis Stimulators") OR ("Angiogenic Factor" AND "Tumor") OR ("Effect*" AND "Angiogenesis") OR ("Factor" AND "Angiogen*") OR ("Factor" A ND "Tumor Angiogenic") OR ("Inducers" AND "Angiogenesis") OR ("Inducing Agents" AND "Angiogenesis") OR ("Stimulating Agents" AND "Angiogenesis") OR ("Stimulators" AND "Angiogenesis") OR ("Tumor Angiogenic Factor") OR ("angiogenesis inducing agent*") OR ("placental angiogenesis fator"))) OR (TITLE-ABS-KEY (("Angiogenesis Modulators") OR ("Modulators" AND "Angiogenesis") OR ("angiogenesis modulating agent*"))) OR (TITLE-ABS-KEY (("Angiogenesis" AND "Physiologic*") OR ("Neovascularization" AND "Physiological") OR ("Physiologic* Angiogenesis") OR ("Physiologic* Neovascularization")))) AND ((TITLE-ABS-KEY (("Healing*" AND "Wound") OR ("Wound Healing*") OR ("granulation" AND "wound") OR ("healing" AND "wound") OR ("repair" AND "wound") OR ("wound granulation") OR ("wound regeneration") OR ("wound repair"))) OR (TITLE-ABS-KEY (cicatrization OR (scar*) OR scarring OR cicatrices OR cicatrix OR ("radiation scar")))) AND ((TITLE-ABS-KEY (("Diabetes Mellitus") OR ("Complications of Diabetes Mellitus") OR ("Diabetes Complication") OR ("Diabetes Mellitus Complication*) OR (" diabetes AND related AND complications ") OR (" diabetes-related AND complication*) OR ("Diabetic Complication*") OR ("Diabetes Insipidus") OR ("Diet" AND "Diabetic") OR ("Prediabetic State") OR ("Scleredema Adultorum") OR ("Glycation End Products" AND "Advanced") OR ("Glucose Intolerance") OR gastroparesis OR diabetes OR diabetic)) OR (TITLE-ABS-KEY (("Diabetic Feet") OR ("Feet" AND "Diabetic") OR ("Foot Ulcer" AND "Diabetic") OR ("Foot" AND "Diabetic") OR ("Diabetic foot") OR ("Foot" AND "Diabetic") OR ("Diabetic Feet") OR ("Feet" AND "Diabetic") OR ("diabetic feet") OR ("diabetic foot syndrome") OR ("diabetic foot ulcer") OR ("foot ulcer" AND "diabetic"))))	**Total:** 10<br><br>**Total after applying the filter:** 10
**Pubmed**<br>January 24, 2023<br>Update: October 19, 2023	("Lab-On-A-Chip Devices"[MeSH Terms] OR "Microfluidics"[MeSH Terms]) AND ("Angiogenesis Inducing Agents"[MeSH Terms] OR "Angiogenesis Modulating Agents"[MeSH Terms] OR "neovascularization, physiologic"[MeSH Terms]) AND ("Wound Healing"[MeSH Terms] OR "Cicatrix"[MeSH Terms]) AND ("Diabetes Mellitus"[MeSH Terms] OR "Diabetic Foot"[MeSH Terms])	**Total:** 01<br><br>**Total after applying the filter:** 01
**Embase**<br>January 24, 2023<br>Update: October 19, 2023	('lab on a chip'/exp OR 'body on a chip'/exp OR 'microfluidics'/exp OR 'microfluidics') AND ('angiogenic factor'/exp OR 'angiogenesis modulator'/exp OR 'angiogenesis'/exp OR 'angiogenesis') AND ('wound healing'/exp OR 'scar'/exp) AND ('diabetes mellitus'/exp OR 'diabetic foot'/exp)	**Total:** 15<br><br>**Total after applying the filter:** 12

**Web of Science** January 24, 2023 Update: October 19, 2023	AB=((“Diabetic Feet”) OR (“Feet” AND “Diabetic”) OR (“Foot Ulcer” AND “Diabetic”) OR (“Foot” AND “Diabetic”) OR (“Diabetic foot”) OR (“Foot” AND “Diabetic”) OR (“Diabetic Feet”) OR (“Feet” AND “Diabetic”) OR (“diabetic feet”) OR (“diabetic foot syndrome”) OR (“diabetic foot ulcer”) OR (“foot ulcer” AND “diabetic”))	**Total:** 02 **Total after applying the filter:** 02
**IEEE Xplore** January 24, 2023 Update: October 19, 2023	("Abstract":Lab.-On-A-Chip Devices) OR ("Abstract":Body on a chip) OR ("Abstract":Microfluidics) AND ("Abstract":Angiogenesis Inducing Agents) OR ("Abstract":Angiogenesis Modulating Agents) OR ("Abstract":Neovascularization Physiologic) AND ("Abstract":Wound Healing) OR ("Abstract":Cicatrix) AND ("Abstract":Diabetes Mellitus) OR ("Abstract":Diabetic Foot)	**Total:** 2511 **Total after applying the filter:** 1445
**CINAHL/ EBSCO** January 24, 2023 Update: October 19, 2023	(((((AB (“Diabetic Feet”) OR (“Feet” AND “Diabetic”) OR (“Foot Ulcer” AND “Diabetic”) OR (“Foot” AND “Diabetic”) OR (“Diabetic foot”) OR (“Foot” AND “Diabetic”) OR (“Diabetic Feet”) OR (“Feet” AND “Diabetic”) OR (“diabetic feet”) OR (“diabetic foot syndrome”) OR (“diabetic foot ulcer”) OR (“foot ulcer” AND “diabetic”)) AND (S1 OR S2)) AND (S3 OR S4 OR S5)) AND (S6 OR S7)) AND (S8 OR S9)) AND (S10 AND S11 AND S12 AND S13 AND S14)	**Total:** 0 **Total after applying the filter:** 0
	**Total number of articles found in the databases:**	**2539**
	**Total number of articles found after applying the filters and removing duplicates:**	**1470**
	**Filters used**: research from the last ten years (2013-2023) and types of articles	
