# Supplementary material for: Advanced microfluidic and 3D cell culture platforms for modeling vascularization in diabetic foot ulcers: A systematic review of translational challenges and perspectives
Source: PLoS One. 2026 Apr 6;21(4):e0328278. doi: 10.1371/journal.pone.0328278 (PMC13052901; doi:10.1371/journal.pone.0328278)
Supplement: S3 File — (PDF) [file pone.0328278.s004.pdf]

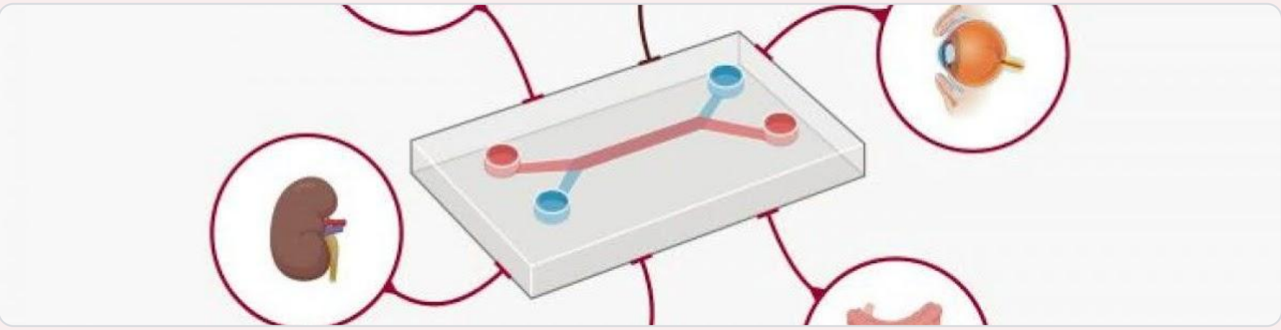

Phase 2 - Full-text reading: Eligibility criteria

PROSPERO Protocol Link: https://www.crd.york.ac.uk/prospero/display_record.php?RecordID=336473

Note: Please re-read the PROSPERO protocol and ensure you are confident about the eligibility criteria before starting the article evaluation. If you have any doubts, consult with the first author.

anakaroline.alms@gmail.com Alternar conta

Não compartilhado

* Indica uma pergunta obrigatória

Reviewer: *

- ◯ Suélia Rosa
- ◯ Ana Karoline Almeida
- ◯ Gustavo Nunes
- ◯ Lindemberg Barreto

Article title *

Sua resposta

Authors of the work *

Sua resposta

Year of publication *

Sua resposta

Published journal *

Sua resposta

Próxima

Limpar formulário

Nunca envie senhas pelo Formulários Google.

Este conteúdo não foi criado nem aprovado pelo Google. Denunciar abuso - Termos de Serviço - Política de Privacidade

Google Formulários

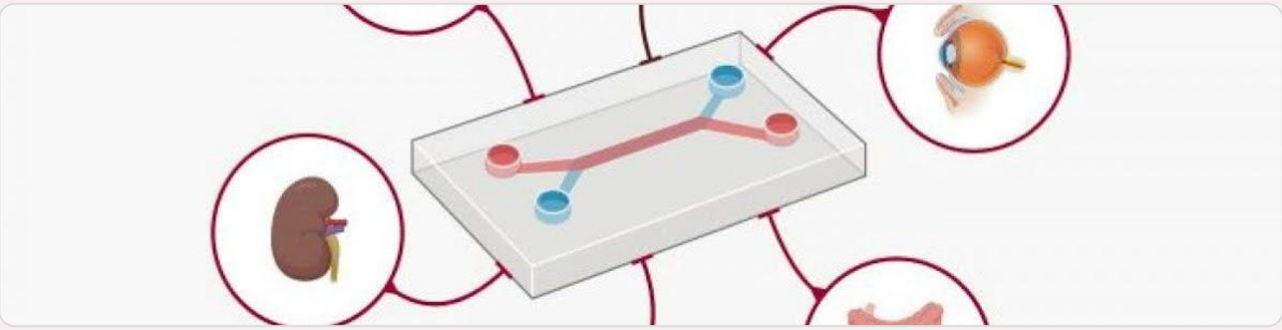

Phase 2 - Full-text reading: Eligibility criteria

anakaroline.alms@gmail.com Alternar conta

Não compartilhado

* Indica uma pergunta obrigatória

Information about the article read in full:

Study design *

- In vitro
- In vivo
- Randomized controlled trial
- In silico
- Review
- Other

Does the study use 3D platforms for in vitro studies? *

- Yes
- No

If the previous answer was yes, what type of platform is used?

- Organoids
- Organ-on-a-chip
- Human-on-a-chip
- Lab-on-a-chip
- Other

Which tissue was used for cell culture? *

Sua resposta

Does the study address systemic circulation, microcirculation, angiogenesis, or vasculogenesis? *

- Yes
- No

Does the study address the scenario of Diabetes Mellitus? *

- Yes
- No
- Outro:

Does the study address diabetic foot wounds? *

- Sim
- Não

Voltar Próxima Limpar formulário

Nunca envie senhas pelo Formulários Google.

Este conteúdo não foi criado nem aprovado pelo Google. Denunciar abuso - Termos de Serviço - Política de Privacidade

Google Formulários

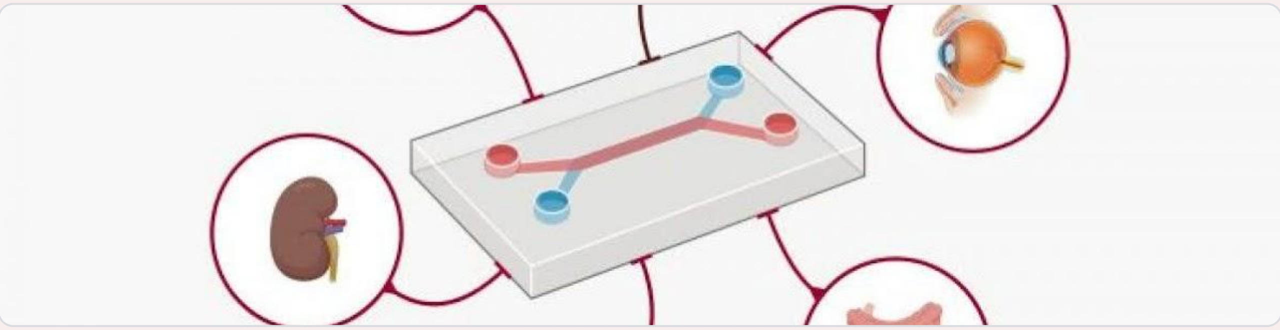

Phase 2 - Full-text reading: Eligibility criteria

anakaroline.alms@gmail.com Alternar conta

Não compartilhado

* Indica uma pergunta obrigatória

Decision on inclusion or exclusion of the study

Based on the eligibility criteria previously described in the PROSPERO protocol, do you decide to include or exclude this study from this systematic review? *

- Include
- Exclude

If you have chosen to exclude, please provide a brief description of the reason in one sentence:

Sua resposta

Voltar Enviar Limpar formulário

Nunca envie senhas pelo Formulários Google.

Este conteúdo não foi criado nem aprovado pelo Google. Denunciar abuso - Termos de Serviço - Política de Privacidade
