# Supplementary material for: Advanced microfluidic and 3D cell culture platforms for modeling vascularization in diabetic foot ulcers: A systematic review of translational challenges and perspectives
Source: PLoS One. 2026 Apr 6;21(4):e0328278. doi: 10.1371/journal.pone.0328278 (PMC13052901; doi:10.1371/journal.pone.0328278)
Supplement: S4 File — (PDF) [file pone.0328278.s005.pdf]

Additional Files 4 - Excluded articles and Reasons for exclusion

Reasons for exclusion	Total
**Reason 01.** The study does not address tissue angiogenesis focused on 3D platforms or organs-on-a-chip	11
**Reason 02.** The study is not relevant or applicable to research focused on Organs-on-a-chip devices	5
**Reason 03.** The work is related to electronic devices in general that are not connected to in vivo 3D platforms	6
**Reason 04.** The article is not linked to studies on chronic wounds or diabetic foot	5
**Reason 05.** It is a literature review	2
**Articles excluded after full-text reading:**	**29**
