# Supplementary material for: Advanced microfluidic and 3D cell culture platforms for modeling vascularization in diabetic foot ulcers: A systematic review of translational challenges and perspectives
Source: PLoS One. 2026 Apr 6;21(4):e0328278. doi: 10.1371/journal.pone.0328278 (PMC13052901; doi:10.1371/journal.pone.0328278)
Supplement: S5 File — (PDF) [file pone.0328278.s006.pdf]

Additional Files 5 - Risk of bias of selected individual studies

---

D1. Were the criteria used to select tissues and cells in the Organ-on-a-chip or 3D cell culture platform described?

D2. Was there an experimental group and a control group in the manufacturing process?

D3. Was identification and authentication of the cell line performed?

D4. Did the study provide a basic morphological description containing cell status and differentiation information?

D5. Was there any non-random allocation of tissues or cells in the Organ-on-a-chip or 3D cell culture platform?

D6. Are the cell layer formation procedure steps clearly described and detailed enough to allow replication?

D7. Did cell fixation and growth have a clearly indicated number of experimental units in each group? And is it the same number (n) that was evaluated in statistical analyses?

D8. Are the pre-established criteria for including or excluding experimental units described during the experiments or analyses?

D9. Were the evaluators of the Organ-on-a-chip assay or 3D cell culture results blinded to the treatment or exposure group?

D10. Was there any loss to follow-up of tissues or cells during the 3D cell culture assay? Was this loss disproportionate between treatment or exposure groups?

D11. Was data collection on the Organ-on-a-chip assay results standardized between treatment or exposure groups?

D12. Is there a detailed description of the Chip Design platform development, including construction and dimensions?

D13. Was submission to the Research Ethics Committee, respective to the type of cell culture, performed?

D14. Are there any confounding factors that could affect the relationship between exposure and the results of the 3D cell culture? If so, were they properly controlled or adjusted in the analysis of the results?

D15. Is there a statement about the presence or absence of conflicts of interest in the study?
